# Individual and Community-level factors associated with early marriage in Zambia: a mixed effect analysis

**DOI:** 10.1186/s12905-023-02168-8

**Published:** 2023-01-17

**Authors:** Million Phiri, Emmanuel Musonda, Liness Shasha, Vincent Kanyamuna, Musonda Lemba

**Affiliations:** 1 Department of Population Studies, School of Humanities and Social Sciences, University of Zambia, Lusaka, Zambia; 2 Demography and Population Studies Programme, Schools of Public Health and Social Sciences, University of the Witwatersrand, Johannesburg, South Africa; 3 Department of Development Studies, School of Humanities and Social Sciences, University of Zambia, Lusaka, Zambia

**Keywords:** Women, Early marriage, Reproductive health, Mixed effect analysis, Zambia

## Abstract

**Background:**

Child marriage has long been a public health concern around the world, because it has the potential to deprive adolescent girls of their sexual reproductive health rights and limits their ability to reach their full potential in life. The prevalence of child marriage has been consistently higher in sub-Saharan Africa than elsewhere. However, fewer studies have explored the influence of both individual and community-level influences on early marriage in sub-Saharan Africa. This study, therefore, examined individual and community-level factors associated with child marriages in Zambia.

**Methods:**

Data came from the Zambia Demographic and Health Surveys (ZDHS) conducted in 2007, 2013–14 and 2018. A pooled weighted sample of 9990 women aged 20–29 years was used in the analysis. Stata software version 17 was used to perform statistical analysis, taking into account complex survey design. The association between individual- and community- level factors and early marital behavior was assessed using multilevel logistic regression models.

**Results:**

The prevalence of child marriage among women aged 20–29 was 44.4 percent (95% CI: 42.1, 46.7) in 2018, declining from 51.5 percent (95% CI: 48.9, 54.0) in 2007. Women with secondary or higher level of education [aOR = 0.36, 95% CI = 0.26–0.49] and [aOR = 0.07, 95% CI = 0.03–0.18] and those whose age at first birth was (15–19 year) or (20–29 years) were associated with less likelihood of experiencing child marriage. Communities with a high percentage of women who gave birth at a young age [aOR = 1.36, 95% CI = 1.15–1.62] were more likely to experience child marriage. Individual and community-level characteristics accounted for 35% of the overall variations in communities' likelihood of experiencing early marriage. Even after controlling for both individual and community-level influences, the intra-class correlation revealed that around 4.5 percent of the overall variations remained unexplained.

**Conclusion:**

Prevalence of child marriage has reduced over the years but is still high in Zambia. Both individual and community- level factors influenced child marriage in Zambia. There is a need to strengthen strategies that keep girls in school to delay their exposure to early sexual debut and child marriage. Designing of reproductive health interventions in the country should consider integration of community factors such as economic insecurity and access to reproductive health information.

**Supplementary Information:**

The online version contains supplementary material available at 10.1186/s12905-023-02168-8.

## Introduction

Child marriage, defined as a legal or informal union between two people before they turn 18 years old, is a practice that disproportionately affects girls and is linked to several unfavorable social and developmental outcomes [1–5]. The United Nations Sustainable Development Goals acknowledges that Child marriage has long been a public health concern around the world [6–8]. This is because it has the potential to deprive adolescent girls of their sexual and reproductive health rights. Furthermore, child marriage can limit their ability to reach their full potential and enjoy their human rights as guaranteed by several international treaties [1, 9–12]. Child marriage remains a burden in developing regions with sub-Saharan Africa having the highest prevalence of 37%, South East Asia at 30% and Latin America at 21% [1, 6, 13, 14]. According to Girls Not Brides in 2018, one out of every five girls is married before the age of 18, with Africa accounting for roughly 67–76 percent of child marriages [7, 15]. Apart from Africa, Asia has a high rate of child marriage, with around 46 percent of women aged 20–24 in South Asia marrying before the age of 18 [16, 17]. Despite global declines in child marriage rates, its persistence in particular places has led to a growing acknowledgment that ending the practice requires a detailed knowledge of the factors that drive it [18, 19]. Several studies on child marriage have revealed a number of socially complex, interconnected, and context-specific variables that vary in importance across and even within nations [1, 11, 13].

The major drivers of child marriage have been conceptualized as follows: poverty and economic factors; lack of opportunity for girls beyond marriage; fear of pregnancy/girls' sexuality; social norms; and a lack of agencies among girls themselves [1, 13, 18, 20–22]. Literature has shown that girls who marry early are more likely to experience violence, abuse, and forced sexual relations because of unequal power relations [23–25]. Young girls are also more vulnerable to sexually transmitted infections (including HIV) [26–28]. Girls’ education, health, and psychologic well-being of females, as well as the health of their offspring, are all negatively impacted by child marriage [29].

Most existing research seeking to explain why child marriage persists has focused on understanding how factors manifest at the individual and household levels. In recent years, there has also been a growing interest in understanding and changing drivers that sustain the practice at the community level [22, 25]. However, few studies have explored how the drivers of child marriage manifest across both micro (individual and household) and macro (community) levels, particularly in sub-Saharan Africa (SSA) [21, 30, 31]. Understanding the intersection of drivers across levels and to what extent drivers work separately or jointly to sustain the practice is critical for designing and implementing effective policies and programs aimed at preventing child marriage. However, we are still learning about factors that influence early marital decision making, particularly about girls’ beliefs and circumstances and about the social context in which they live [32–34].

Even though literature shows that the environment has a significant impact on marital and reproductive health behaviour of young individuals, mainly due to peer pressure and other social factors, no study has attempted to examine both individual and community level factors associated with child marriage in Zambia. An earlier study by Mulenga and others [35], conducted in Zambia found that residence, age at first sex, education level of women and their partners, and family size had a significant influence on prevalence of child marriage. The study, however, ignored the influence of community-level factors on child marriage. There is a paucity of knowledge on how community-level factors influence early marriage in Zambia. In view of this, we conducted this study to bridge the knowledge gap that exists in the literature. Examining both individual and community-level factors associated with child marriage is an important step to inform relevant government and non-governmental organizations to have an in-depth understanding of factors that explain why girls fall into a trap of child marriages in Zambia.

Despite many efforts by government and stakeholders to address social and economic factors that predispose young girls to marry early, the prevalence of child marriage is still high in Zambia. In 2013, 31.4% of women aged 20–24 reported to have been married before age 18 [1, 9, 10]. The prevalence is significantly higher in rural areas than in urban areas [9, 36, 37]. We therefore conducted this study to investigate individual and community-factors associated with child marriage in Zambia. The study also sought to establish if there are community-level variations in the prevalence of child marriage. There is increasing evidence in the literature that the environment has a significant impact on young people's reproductive behaviour [38–42]**.** Therefore, the application of the multilevel analysis model in this study allowed for in-depth analysis of the effects of both individual and community variables on women’s decision about early marriage. Our study has provided an opportunity to generate information useful for shaping and redesigning of existing policies and interventions aimed at eliminating early marriage in the country. The findings might inform reproductive health policies and programming in other parts of Sub-Saharan Africa.

## Methods

### Data source

**Fig. 1:**
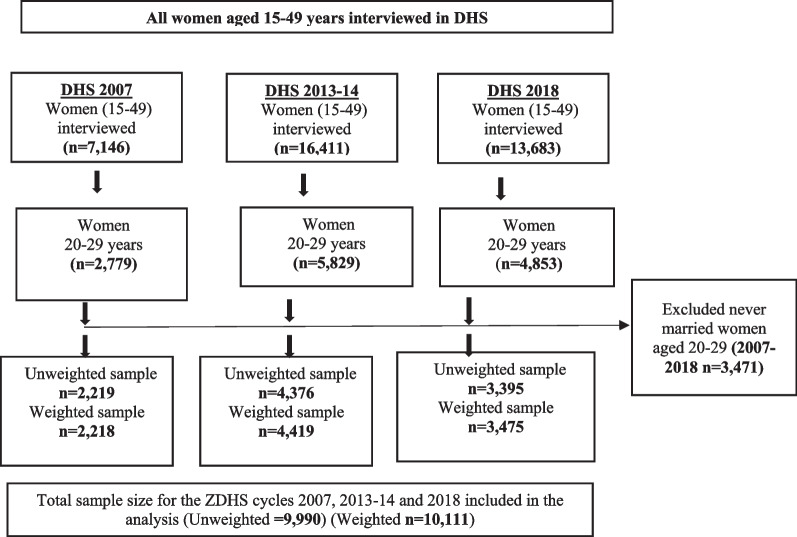
Sample selection and inclusion criteria

Secondary data from the Zambia Demographic and Health Survey (ZDHS) conducted in 2007, 2013 and 2018 was used [43]. Specifically, the study used the women’s individual recode files (IR) which contain the responses of women aged 15–49 who were enrolled in surveys. The Demographic and Health Survey (DHS) is a nationwide cross-sectional survey that is usually carried out across low-and middle-income countries every five-years [44] and collects data on several indicators such related to demographic and health of a country. The DHS has been an essential source of country level data on issues surrounding sexual and reproductive health indicators in low-and middle-income countries as it gathers data on several indicators such as marriage, sexual-activity, fertility, fertility-preferences and family-planning [44]. Stratified, two-stage sampling approach is usually employed in selecting the sample for the DHS. A pooled sample of 9990 women aged 20–29 years, who were ever-married prior to survey and had complete information on reported age at first marriage were included in the analysis. The age group 20 to 24 years old is the typical age range for researching child marriage among women who have ever been married [1, 11, 17]. We used the broader age range of the ever-married women 20 to 29 years old for analyses of the data samples because the age sample for the group 20–24 years was not sufficient for our analysis. Because age at first marriage was measured retrospectively, we excluded all teenage women aged 15–19 years from the analysis. This is due to the fact that not all members of this cohort had a chance to experience child marriage as they had not yet completed childhood age. The selection criteria for the study sample size for the three DHS’s is described in Fig. 1.

### Measures

#### Outcome measure

The outcome variable for this study was age at marriage. Age at first marriage is defined as “age at which woman or a man was first married or stated cohabiting with partner” usually age at first marriage is presented as; less than 18 years or 18 years and above [8, 9, 35]. During the DHS survey, all women who reported being ever married prior to the survey were asked to state the age at which they got married or started cohabiting with a partner. The variable was collected and recorded as continuous data. To facilitate binary analysis, we then recoded the variable into two categories: (i) ‘less than 18 years’ and; (ii) 18 years or above. A binary outcome variable was then classified as “0” representing age at first marriage/cohabitation of 18 years or above and “1” representing age at first marriage below 18 years, which was treated as child marriage.

#### Independent variables

Based on exiting literature [30, 34, 35, 45], a number of explanatory variables were selected, these included: age of a woman; age at first sex; education; literacy; residence; region; wealth status; employment status; exposure to family planning messages; age at first birth; gave birth in the last five years; age of partner; education of partner; and employment of partner. These variables were grouped into individual and community-level variables.

#### Individual level factors

Individual-level factors included age of a woman categorized as [20–29]; education level (none, primary, secondary and higher); literacy (illiterate and literate); age at first sex (less than 15, 15–19, 20–24 and 25–29); age at firth birth (less than 15, 15–19 and 20–29); age of a partner at the time of the survey (less than 25, 25–29, 30–34, and 35 +); wealth status (poor, middle and rich). Other individual variables included employment status (not working and working); education level of partner (none, primary, secondary, and higher); partner’s employment status (not working and working); gave birth last five years (no and yes); media exposure (no and yes); and desired family size (less than 4 children, 4–5 and 6 + children).

#### Community-level factors

The aggregation of socioeconomic and demographic characteristics (education, employment, wealth status, age at first birth) and behaviour-related factors (fertility desire, exposure to FP messages) from individual-level to community-level was done to study these variables at the community or neighbourhood level. These community variables were chosen based on their significance in previous research [21, 30]. A community was defined as the primary sampling unit (i.e., cluster) of the ZDHS’s. Household wealth, employment, women’s education, age at first birth, ideal number of children, and exposure to media FP messages were aggregated to a sampling unit to generate community level. The community-level factors, except for residence, were aggregated individual-level variables at the cluster level measured as average proportions classified into low, medium, and high levels for each variable for easy interpretation. The following categorisation was used to group the percentile into three discrete categories (low = “0–49 percent”; medium = “50–75 percent”; high = “75–100 percent”). A number of studies guided the construction of the indices and community variables used in this study [22, 25, 40, 45–47].

### Statistical analysis

Data analysis was done at three levels: descriptive, bivariate and multilevel using Stata version 17 software, with 5% level of significance. At the descriptive level, percent distributions of outcome indicators were presented. At the bivariate level, cross-tabulations with chi-square tests were used to analyse the association between child marriage and the selected independent variables. In order to assess the effects of several identified individual and community-level factors on child marriage in Zambia, a two-level multilevel binary logistic regression model was applied on a pooled data for all the three surveys phases. First level involved analysing data at the individual level and the second involved analysis at community level. The “melogit” command was used in Stata software to account for the clustering of the outcome variable within and across sampling clusters of the survey design. Adjusted odds ratios (aOR) with corresponding 95% confidence intervals (CI) were reported. Four multilevel logistic models were estimated. Model 1 included the outcome variable only in order to test the random variability in the intercept. Model 2 included the individual-level variables to examine women’s characteristics on early marriage experience while Model 3 examined the effect of community-level characteristics only; model 4 included both the individual and community-level factors. All covariates were included in the multilevel analyses regardless of level of significance at bivariate analysis. This is because all the variables in our study conceptual framework have been reported to significantly influence child marriage in prior studies [30, 34, 35, 45].

The intra-class correlation (ICC) was used to understand variations of relationships between communities and the relative effect of community-level variables. ICC provides information on the share of variance at each level. The latent method was used to calculate the PVC at each level. It assumes a threshold model, approximating the level 1 variance by ${\pi }^{2}/3(\approx 3.29)$ [40, 47, 48]. To explain the heterogeneity in the probabilities of early marital experience, the Proportional Change in Variance (PCV) was computed for each model compared to the empty model. The PCV provided information on the share of variance for each model relative to model I. Aikake Information Criteria (AIC) were used to compare models and measure goodness of fit [40, 47].The model with the lower Aikake Information Criteria (AIC) was considered being a better fit for the data. To assess multicollinearity among independent factors, the variance inflation factor (VIF) was used. There were no concerns with multicollinearity in any of the variables (all VIF < 5). The variance inflation factor values are presented in Additional file 1: Table 1.

### Ethical approval

The data analysed in this study is available in the public domain at (https://dhsprogram.com/) Permission to use the data was obtained from the DHS program. All datasets used in this study did not contain any personal identification information from survey participants. The original Zambian DHS Biomarker and survey protocols were approved by Tropical Disease and Research Center (TDRC) and the Research Ethics Review Board of the Center for Disease Control and Prevention (CDC) Atlanta.

## Results

### Sample description

A total of 9990 women were included in the analysis across the three ZDHS surveys. The majority, 44 percent, were from the 2013/14 ZDHS, while 34 percent were from the 2018 survey and 21 percent from the 2007 survey. Table 1 shows the distribution of women included in the analysis by background characteristics across the survey years. The distribution of respondents across ages shows that most of them (above 50 percent) were aged between 25 and 29 years in all the three surveys years. Rural and urban distribution showed that in all three survey years, 2007, 2013 and 2018, the majority of respondents were from rural areas (63 percent, 57 percent and 59 percent respectively). The provincial distribution shows that Lusaka had the highest proportion of women across the two survey years 2013 and 2018 (19 percent and 19 percent respectively) while Copperbelt (16 percent) had the highest proportion of women for the survey year 2007. Regarding the highest level of education majority of respondents had attained only primary school level education across the three survey years 2007, 2013 and 2018 (59 percent, 49 percent and 46 percent respectively). In terms of employment status, 56 percent in 2007, 54 percent in 2013 and 52 percent in 2018 were working.

**Table 1:** Percent distribution of background characteristics of ever married young women (20–29 years), 2007–2018 DHS, Zambia

Background characteristics	DHS 2007 (N = 2219)	DHS 2013–14 (N = 4376)	DHS 2018(N = 3395)
	%	**%**	**%**
*Age*			
20–24	45.5 [43.0,48.0]	43.3 [41.6,45.0]	47.4 [45.5,49.3]
25–29	54.5 [52.0,57.0]	56.7 [55.0,58.4]	52.6 [50.7,54.5]
*Residence*			
Urban	37.0 [31.1,43.3]	42.9 [38.9,46.9]	40.7 [35.3,46.3]
Rural	63.0 [56.7,68.9]	57.1 [53.1,61.1]	59.3 [53.7,64.7]
*Province*			
Central	10.4 [7.4,14.4]	9.3 [7.5,11.6]	8.3 [6.4,10.6]
Copperbelt	15.9 [11.2,21.9]	15.4 [12.4,19.1]	13.1 [10.1,16.8]
Eastern	14.3 [10.5,19.2]	12.8 [10.5,15.5]	14.6 [11.6,18.2]
Luapula	7.5 [5.3,10.5]	7.8 [6.3,9.7]	7.8 [6.0,10.1]
Lusaka	14.9 [10.7,20.2]	18.6 [15.3,22.4]	18.6 [14.7,23.2]
Muchinga	-	5.7 [4.5,7.1]	5.9 [4.3,7.9]
Northern	15.1 [11.2,20.1]	8.1 [6.5,10.1]	9.1 [6.9,11.8]
North-Western	5.4[3.7,7.9]	4.3 [3.4,5.4]	4.3 [3.0,6.2]
Southern	10.6 [7.7,14.4]	12.9 [10.6,15.7]	13.8 [9.0,20.4]
Western	5.9 [4.2,8.3]	5.1 [4.0,6.4]	4.6 [3.5,6.1]
*Education level*			
None	10.5 [8.9,12.5]	8.7 [7.6,9.9]	6.4 [5.3,7.8]
Primary	58.8 [55.6,61.9]	49.4 [47.2,51.7]	45.6 [43.2,48.1]
Secondary	27.2 [24.4,30.1]	38.0 [35.8,40.3]	44.6 [42.0,47.1]
Higher	3.5 [2.5,5.0]	3.9 [3.1,4.8]	3.4 [2.6,4.4]
*Wealth status*			
Poor	38.9 [34.2,43.8]	41.2 [38.1,44.4]	41.9 [37.7,46.1]
Middle	20.1 [17.4,23.0]	18.4 [16.7,20.2]	18.8 [16.8,20.9]
Rich	41.0 [35.4,46.9]	40.4 [36.8,44.1]	39.4 [34.9,44.0]
*Employment status*			
Not working	43.7 [40.6,46.9]	46.3 [43.9,48.6]	48.2 [45.5,51.0]
Working	56.3 [53.1,59.4]	53.7 [51.4,56.1]	51.8 [49.0,54.5]
*Age at first birth*			
Less than 15 years	4.3 [3.5,5.4]	4.2 [3.6,5.0]	4.5 [3.7,5.3]
15–19 years	67.4 [65.1,69.6]	69.4 [67.7,71.0]	68.2 [66.1,70.3]
20–29 years	28.3 [26.1,30.6]	26.4 [24.8,28.0]	27.3 [25.4,29.4]
*Media family planning exposure*			
No	81.0 [77.3,84.1]	80.1 [77.9,82.2]	88.5 [86.7,90.1]
Yes	19.0 [15.9,22.7]	19.9 [17.8,22.1]	11.5 [9.9,13.3]

Table 1 further shows that in all survey years 2007, 2013 and 2018, most of the women had their first birth in the age group 15–19 (67 percent, 69 percent and 68 respectively). Majority of the women, ranging from 80 to 89 percent, had no exposure to family planning messages all survey years.

Table 2 shows the prevalence of child marriage according to different background characteristics of women. The trends and pattern show that over the years 2007 to 2018, the prevalence of child marriage reduced from 52 to 44 percent in Zambia. Bivariate analysis reveals that many socio-economic and demographic variables were consistently associated with child marriage (*p* < 0.001). The percentage of women who experienced child marriage was high in all the survey years among those aged 20–24 compared to those aged 25–29.

**Table 2:** Percent distribution of bivariate analysis of child marriage prevalence among ever married women (20–29 years) by background characteristics, 2007–2018 DHS, Zambia

Background characteristics	DHS 2007 (N = 2219)	DHS 2013–14 (N = 4376)	DHS 2018 (N = 3395)
	% (CI)	% (CI)	% (CI)
Age	***	*	**
20–24	56.5 [48.8,54.1]	49.3 [46.6,52.1]	48.1 [45.0,51.4]
25–29	47.2 [43.9,50.6]	44.7 [42.0,47.5]	41.0 [37.9,44.2]
Residence	***	***	***
Urban	41.7 [36.7,46.8]	39.0 [35.5,42.7]	34.1 [30.1,38.3]
Rural	57.2 [54.4,60.0]	52.5 [50.0,54.9]	51.5 [49.1,53.8]
Province	***	***	***
Central	49.7 [43.2,56.3]	48.5 [43.0,54.0]	44.8 [39.5,50.2]
Copperbelt	46.1 [37.1,55.4]	38.4 [32.5,44.6]	31.7 [25.0,39.3]
Eastern	61.6 [56.4,66.6]	57.6 [51.5,63.4]	56.6 [51.7,61.5]
Luapula	62.2 [55.5,68.5]	47.9 [41.5,54.3]	45.0 [40.4,49.7]
Lusaka	43.1 [35.7,50.8]	42.2 [36.1,48.5]	36.6 [30.4,43.3]
Muchinga	–	54.3 [48.2,60.2]	52.0 [45.5,58.3]
Northern	58.2 [52.3,63.9]	55.3 [49.8,60.7]	54.3 [49.0,59.6]
North-Western	56.8 [47.1,65.9]	44.3 [38.0,50.8]	34.6 [26.0,44.2]
Southern	45.7 [39.0,52.6]	48.1 [43.0,53.3]	49.1 [43.5,54.7]
Western	39.9 [31.0,49.5]	32.2 [25.2,40.0]	37.5 [30.6,44.8]
Education level	***	***	***
None	67.7 [61.6,73.3]	62.8 [57.2,68.1]	65.1 [57.8,71.8]
Primary	60.5 [57.4,63.5]	58.9 [56.4,61.3]	58.7 [55.9,61.5]
Secondary	31.6 [27.7,35.9]	31.6 [28.7,34.7]	29.9 [27.1,32.9]
Higher	5.2 [1.7,15.2]	3.6 [1.5,8.4]	1.9 [0.7,5.1]
Age at first Sex	***	***	***
Less than 15	66.4 [59.7,72.4]	66.4 [60.6,71.8]	69.8 [65.3,73.9]
15–19	48.1 [44.6,51.7]	41.7 [39.1,44.4]	43.0 [40.5,45.5]
20–24	6.7 [3.4,12.9]	2.2 [0.9,5.0]	7.0 [1.7,7.4]
Age at firth birth	***	***	***
Less than 15	82.1 [71.8,89.2]	81.4 [73.9,87.0]	85.9 [79.3,90.6]
15–19	69.0 [66.0,71.8]	61.4 [59.0,63.6]	58.7 [56.3,61.2]
20–29	11.5 [8.8,14.9]	8.5 [6.6,10.9]	6.5 [4.8,8.8]
Wealth status	***	***	***
Poor	59.3 [55.9,62.7]	55.3 [52.6,58.0]	53.2 [50.4,55.9]
Middle	56.2 [51.4,60.9]	49.9 [46.0,53.7]	50.3 [46.3,54.4]
Rich	41.7 [37.0,46.5]	36.5 [32.8,40.3]	32.3 [28.4,36.3]
Employment status	Ns	Ns	Ns
Not working	48.9 [45.1,52.8]	45.3 [42.3,48.3]	44.0 [40.3,47.7]
Working	53.4 [50.0,56.8]	47.9 [45.2,50.7]	44.4 [41.1,47.8]
Education level of partner	***	***	***
None	68.2 [59.1,76.1]	65.7 [58.9,71.8]	65.9 [55.7,74.8]
Primary	58.8 [55.6,62.0]	58.9 [55.8,61.9]	55.7 [52.0,59.2]
Secondary	46.8 [42.9,50.8]	41.4 [38.8,44.1]	38.9 [35.7,42.2]
Higher	19.9 [13.4,28.4]	12.9 [9.1,18.0]	14.5 [10.1,20.5]
Employment status of partner		Ns	***
Not working	–	39.4 [25.5,55.4]	54.7 [48.4,60.8]
Working	–	47.0 [44.9,49.1]	42.5 [39.8,45.3]
FP Media exposure	***	***	***
No	55.2 [52.8,57.7]	50.4 [48.3,52.5]	46.5 [44.2,48.8]
Yes	35.4 [29.1,42.1]	31.8 [27.2,36.7]	28.5 [23.2,34.4]
Desired family size	***	***	***
0–3	32.5 [28.0,37.3]	36.3 [32.5,40.3]	34.6 [30.4,39.1]
4–5	53.0 [49.6,56.3]	45.6 [42.8,48.3]	43.6 [40.7,46.5]
6 +	64.6 [60.3,68.7]	57.9 [54.2,61.5]	56.4 [52.6,60.2]
Total	51.5 [48.8, 54.1]	46.5 [44.6, 48.8]	44.4 [42.1,46.7]

^***^p < 0.001; **p < 0.01; *p < 0.05; *Ns* Non-significant

Child marriage has been consistently high among women living in rural areas than among their counterparts living in urban areas. Results show that there has been a large decline in the prevalence of child marriage from 42 to 34 percent in urban areas compared decline observed in rural areas (57 percent to 52 percent) in the period 2007–2018. The provincial prevalence of child marriage in 2018 ranged from 32 percent on the Copperbelt to 57 percent in Eastern province. The prevalence of child marriage was high among women with no formal education across the three survey years (*p* < 0.001). Prevalence of child marriage in Zambia has been high among women with no formal or primary level of education).

Across all the three survey years, the prevalence of child marriage was observed to be high among women whose age at first sexual debut was below the age of 15. This prevalence increased from 66 to 70 percent in the period 2007–2018. Furthermore, our study results show a significant decline in prevalence among women coming from all income groups between 2007 and 2018. There was a slight decline in child marriage among women whose partner’s level of education was primary or secondary from 59 to 56 percent and 47 percent to 39 percent between 2007 and 2018, respectively.

Across the three surveys, the prevalence of child marriage was high among women who had no exposure to family planning messages: 55 percent, 50 percent and 47 percent, respectively. The study results also reveal that young women who preferred a large family size were more likely to marry early compared to their counterparts who preferred a low family size.

### Factors associated with experience of early marriage

#### Modelling approaches (fixed effects)

Table 3 displays the measures of association from the multilevel binary logistic regression model of association between child marriage, individual, and community-level factors. Results of model IV which accounted for both individual and community-level factors, revealed that women with secondary education or higher were less likely to experience child marriage compared to those with no education. Women whose age at first sex was in age groups 15–19 and 20–24 years and those whose age at first birth was in the age group 15–19 and 20–29 years were equally less likely to experience child marriage compared to women who initiated sexual debut or had a first birth at before age 15. Furthermore, women whose partners’ education was secondary or higher were less likely to experience early marriage compared to those whose partners had no formal education. Women from communities with a moderate percentage of women belonging to poor households, from communities with a high percentage of women giving birth at a young age were more likely to experience child marriage compared with their defined counterparts who belonged to poor households and those from communities with low percentage of women giving birth at a young age, respectively. Although nearly all the covariates in the model were significant in the univariable models, very few remained significant after adjustment in the full multivariable model (Model IV).

**Table 3:** Multilevel parameter estimates and adjusted odds of child marriage prevalence, DHS 2007 -2018

Variable	Model I	Model II aOR (95%CI)	Model III aOR (95%CI)	Model IV aOR (95%CI)
Individual factors				
Age				
20–24		1		1
25–29		1.02(0.88–1.18)		1.02(0.88–1.18)
Education level				
None		1		1
Primary		0.82(0.62–1.09)		0.81(0.61–1.08)
Secondary		0.36***(0.26–0.50)		0.36***(0.26–0.49)
Higher		0.07***(0.03–0.18)		0.07***(0.03–0.18)
Wealth status				
Poor		1		1
Middle		1.11(0.93–1.34)		1.10(0.91–1.34)
Rich		1.11(0.90–1.37)		1.15(0.89–1.48)
Employment status				
Not working		1		1
Working		1.02(0.85–1.21)		1.02(0.85–1.23)
Age at first Sex				
Less than 15		1		1
15–19		0.55***(0.45–0.69)		0.56***(0.45–0.70)
20–29		0.12***(0.07–0.21)		0.12***(0.06–0.21)
Age at firth birth				
Less than 15		1		1
15–19		0.57**(0.39–0.83)		0.57**(0.39–0.83)
20–29		0.04***(0.03–0.07)		0.04***(0.03–0.07)
Education level of partner				
None		1		1
Primary		0.92(0.66–1.28)		0.93(0.66–1.29)
Secondary		0.71*(0.50–0.99)		0.71*(0.50–0.99)
Higher		0.55*(0.33–0.90)		0.56*(0.34–0.92)
Employment status of partner				
Not working		1		1
Working		0.96(0.69–1.34)		0.97(0.70–1.35)
Media exposure				
No		1		1
Yes		1.05(0.83–1.33)		1.05(0.83–1.33)
Desired family size				
Less than 4		1		1
4–5		1.08(0.88–1.32)		1.07(0.87–1.32)
6 +		1.28*(1.00–1.64)		1.28(0.99–1.64)
Factors at community level				
Place of residence				
Urban			1	1
Rural			1.67***(1.45–1.91)	0.98(0.78–1.22)
Community poverty				
Low			1	1
Medium			1.02 (0.90–1.18)	1.29*(1.06–1.57)
High			0.90 (0.76–1.07)	0.99(0.77–1.27)
Community education				
Low			1	1
Medium			1.34***(1.18–1.51)	1.00(0.84–1.21)
High			1.50***(1.18–1.75)	0.98(0.79–1.22)
Community employment				
Low			1	1
Medium			1.00(0.89–1.14)	0.94(0.79–1.22)
High			1.06(0.87–1.19)	1.11(0.93–1.33)
Community young age at first birth				
Low			1	1
Medium			1.37**(1.22–1.54)	1.16(0.98–1.37)
High			1.72**(1.53–1.94)	1.36***(1.14–1.62)
Community FP media exposure				
Low			1	1
Medium			1.04(0.91–1.19)	0.97(0.80–1.16)
High			1.00(0.87–1.15)	1.06(0.86–1.29)
Community fertility preference				
Low			1	1
Medium			1.07(0.94–1.21)	1.03(0.86–1.24)
High			1.09(0.83–1.25)	1.01(0.83–1.23)
Random effects				
Variance (CI)	0.23(0.17–0.30)	0.18(0.11–0.30)	0.10 (0.06–0.15)	0.15(0.09–0.27)
ICC (%)	6.4	5.3	2.9	4.5
PCV (%)	Ref	22.7	56.5	35.0
Model fitness				
Log likelihood	− 6910.4	− 3358.6	− 6752.9	− 3344.8
AIC	13,824.9	6757.3	13,535.8	6755.6
N	9990	9990	9990	9900

Model I contains no explanatory variables; Model II includes individual-level factors only; Model III includes community-level factors only; Model IV includes both individual-level and community-level factors

*aOR* adjusted odds ratio, *CI* Confidence internal, *ICC* Intraclass correlation coefficient, *PCV* Proportional change in variance, *AIC* Akaike information criterion

*** *p* < 0.001; ** *p* < 0.01; * *p* < 0.05

#### Modelling approaches (random effects)

Measures of variation for child marriage experience are presented in Table 3. In the null model, the use of multilevel modelling was justified by the significant variation in prevalence of child marriage (σ2 = 0.23, 95% CI 0.17–0.30). The ICC for the child marriage prevalence was 6.4% suggesting that variation in experience of child marriage across clusters may be attributed to other unobserved community-level characteristics. The final model (Model IV) reveals significant variances, showing the effects of community heterogeneity. Additionally, 35 percent of the variance in the odds of experiencing child marriage across communities were explained by both individual and community-level factors, as indicated by the Proportional Change in Variance (PCV) in model IV.

## Discussion

This study sought to analyse the influence of individual and community-level factors that explain child marriage in Zambia. The study applied a multilevel logistic regression models on the pooled 2007–2018 Zambia Demographic and Health Surveys to better understand the factors that explain child marriage among women in Zambia. Disparities in experience of early marriage have been observed among different sociodemographic strata and understanding factors associated with early marriage in Zambia has a significant implication on strengthening sexual reproductive health policies and programmes to further reduce the prevalence of child marriage.

Our study reveals that the proportion of child marriage among women was 44.4 percent in 2018, declining from 51.5 percent in 2007. This decline could be attributed to the design and implementation of the national multi-sectoral strategy to end child marriage, which was launched in 2016 [9]. The study established that individual factors (education level of a woman, partners’ education, age at first sexual debut, age at first birth and desired family size) and community-level factors (poverty level, young age at first birth) were significantly associated with early marriage in Zambia. The high prevalence of child marriage remains a public health and social concerns to achievement of sustainable development goals on improving maternal health and women’s education. These results suggest urgent attention for strengthening sexual reproductive health policies and programming in Zambia. Conversely, the study has revealed that residence, household wealth status, employment status and other community-level factors had no effect on experience of child marriage among young women in Zambia. A similar study by Zegeye [45] found that apart from region, all community-level factors were not associated with an experience of child marriage in Mali.

In this analysis, women with secondary or higher level of were less likely to experience child marriage. This suggests that increasing schooling opportunities for women have a significant bearing on reducing the prevalence of child marriages in Zambia, because educated women have the potential to make an informed decision about marital behavior because of easy access to appropriate reproductive health information. Our finding is consistent with similar studies conducted in Tanzania, Burkina Faso, Ethiopia and Nigeria [21, 30, 49] which also reported education as a significant factor in reducing exposure to early marriage. Additionally, women whose partners had secondary or higher education are less likely to experience early marriage compared to those whose partners had a lower level of education. This finding is similar to what was reported in previous studies [45, 50].

Our findings revealed that women who delayed their first sexual debut were less likely to experience early marriage compared with those started having sexual intercourse early. This finding is consistent with a study conducted in Tanzania and Burkina Faso in which age at first sex was positively associated with early marriage [21, 30]. Sexual behavior of women has shown to influence marital behavior in other settings. For instance, in Congo, women who started sexual debut before age 16 were three and a half times more at risk of early marriage than those who started having sexual intercourse after age 17 [51]. Our study found that women who started childbearing after age 14 were less likely to marry early. The variations in experience of early marriage according to different sexual reproductive behavior observed in this study underscore the need to strengthen comprehensive sexual reproductive education and contraceptive services in primary and secondary education curriculum to reduce child marriage in Zambia effectively.

The effects of community-level characteristics on child marriage have been well-documented [21, 45]. Findings from the current analysis revealed that women from communities with a moderate percentage of women belonging to poor households and from communities with a high percentage of women giving birth at a younger age were more likely to experience child marriage compared to their defined counterparts. Women from poor households may have inadequate access to sexual reproductive health information and services. Additionally, these women may also have less access to education, hence being less likely to comprehend health messages leading to a low understanding of consequences of early marriage and reduced demand of reproductive health services [33, 52]. Similarly, women from well-to-do communities are expected to better have chances of accessing education and sexual reproductive health information. As such, these women are empowered to make an informed decision about health and social consequences of early marriages.

Differences in experience of child marriage were observed according to distinct individual and community factors. Therefore, increasing access to education for female adolescents and women and strengthening sexual reproductive programme interventions will be key to addressing the problem of child marriage in Zambia. As evidenced by the results, employed women were less likely to have experienced child marriage, suggesting that empowering women may go a long way in addressing child marriage. There could be unobserved or unmeasured community-level factors that influenced early marital behavior. This suggests that there could be factors operating at the community-level, not included in the current analysis, which may be associated with early marital behavior in Zambia. These may include, but are not limited to, cultural differences between communities (that may ultimately influence child marriage), and community outreach, engagement, and mobilization efforts. Therefore, sexual reproductive programs need to be embedded in early school curriculum and a thorough community profiling. Furthermore, interventions to curb child marriage require approaches that will strengthen community engagement among relevant stakeholders such as civic leader, traditional leaders, community leaders and religious institutions.

Although the study has provided useful findings that have the potential to inform strengthening of existing sexual reproductive policies and programming targeting at changing marital behaviour among adolescents and women in Zambia. Designing of tailor-made interventions to address the problem will require a detailed decomposition analysis of both individual and community-level factors to delineate factors that have contributed to the observed reduction in prevalence of child marriage.

### Study strengths and limitations

The study had a number of limitations. First, because of the cross-sectional nature of the DHS data, causality cannot be inferred from this study. Second, the outcome of interest child marriage was measured retrospectively using the reported age at first marriage for the ever-married women. But the independent factors are with reference to the time when the survey was conducted, meaning that there is a possibility of a variance between the factors at the time of marriage and those at the time of the survey. There is also a possibility of recall bias, since the DHS participants were asked to report events that happened in the past. Since the study comprised a nationally representative sample of Zambian women, the current findings can apply to the entire population of women in Zambia. The hierarchical nature of the DHS dataset allowed for exploration of community effects, which may have an influence on sexual and reproductive health and family planning programming in Zambia. The study assessed a wide range of factors to strengthen the associations observed.

## Conclusion

The study has established that the prevalence of child marriage is still high in Zambia, even though there has been a reduction in the trends over the years. In Zambia, we have observed that the factors that influence child marriage operate at both individual and community-level level. Sexual and reproductive health programmes should be strengthened, especially among communities with less access to education in order to improve reproductive health outcomes, such as age at first sexual intercourse and age at first birth among women. Further research is needed to have a better understanding of some findings reported in this study, as well as to learn more about the socio-cultural and religious influences that may explain some of the unaccounted for community effects on child marriage.

## Supplementary Information

**Additional file 1. Supplementary file Table 1:** Results of Multicollinearity Test.

## Data Availability

Datasets used in our study are publicly available at DHS program website (https://dhsprogram.com/).

## Abbreviations

CI: Confidence interval
EA: Enumeration area
FP: Family planning
SDG: Sustainable development goal
SRH: Sexual reproductive health
SSA: Sub-Saharan Africa
UNFPA: United Nations Population Fund
UNICEF: United Nations Children Fund
USAID: United States Aid for International Development
WHO: World Health Organisation
ZDHS: Zambia Demographic and Health Survey
