# Supplementary material for: Individual and Community-level factors associated with early marriage in Zambia: a mixed effect analysis
Source: BMC Womens Health. 2023 Jan 17;23:21. doi: 10.1186/s12905-023-02168-8 (PMC9843915; doi:10.1186/s12905-023-02168-8)
Supplement: Supplementary file 1 — Additional file 1. Supplementary file Table 1: Results of Multicollinearity Test. [file 12905_2023_2168_MOESM1_ESM.docx]

| Supplementary file Table 1: Results of Multicollinearity Test | |
| --- | --- |
| **Variable** | **VIF** |
| **Age of a woman** | 1.44 |
| **Residence** | 2.15 |
| **Women's Education level** | 1.60 |
| **Women's Age at first Sex** | 1.29 |
| **Age at firth birth** | 1.34 |
| **Wealth Status** | 2.63 |
| **Women's Employment Status** | 1.15 |
| **Education level of partner** | 1.52 |
| **Employment status of partner** | 1.05 |
| **Gave birth last five years** | 1.07 |
| **Desired family size** | 1.24 |
| **Media exposure to family planning messages** | 1.33 |
| **Community poverty** | 2.23 |
| **Community education** | 1.75 |
| **Community employment** | 1.17 |
| **Community age at first birth** | 1.06 |
| **Community access media FP exposure** | 1.67 |
| **Community fertility preference** | 1.38 |
| VIF; variance inflation factor |  |
